# Seclusion and mechanical restraint in the wake of the COVID-19 pandemic: an increased use in mental health settings

**DOI:** 10.3389/fpsyt.2024.1428599

**Published:** 2024-10-04

**Authors:** Marie-Hélène Goulet, Christine Cassivi, Catherine Hupé, Francelyne Jean-Baptiste, Alexandre Dumais

**Affiliations:** ^1^ Nursing Faculty, Université de Montréal, Montréal, QC, Canada; ^2^ Centre de recherche de l’Institut universitaire en santé mentale de Montréal, Montréal, QC, Canada; ^3^ Nursing Excellence Center, Integrated Health and Social Services Centres (CIUSSS)-Est-de-l’Île-de-Montréal, Montréal, QC, Canada; ^4^ Departement of Psychiatry and Addictology, Faculty of Medicine, Université de Montréal, Montréal, QC, Canada

**Keywords:** seclusion, restraint, coercion, prevalence, COVID-19, psychiatry

## Abstract

**Purpose:**

COVID-19 pandemic-related restrictions have significantly changed the environment and the delivery of direct care in all health services, including psychiatric hospitals. The aim of the study is two-fold: 1) to retrospectively assess the incidence of seclusion and mechanical restraint in a Quebec mental health hospital over 4 years; and 2) to assess the impact of the COVID-19 pandemic on their incidence.

**Methods:**

We conducted a retrospective study based on medical records from a Quebec mental health hospital collected (a) from April 2016 to March 2019), (b) from April 2019 to March 2020 (pre-COVID onset), and (c) from April 2020 to March 2021 (post-COVID onset). Descriptive statistics, chi square tests, Mann-Kendall test and Welch’s t-test were performed.

**Results:**

Following several years during which the use of restrictive measures remained stable, we have noted a significant increase within the first year following the COVID-19 pandemic. This increase can be seen in the use of both seclusion and restraints, which have risen 205% and 170% respectively.

**Conclusion:**

There are a multitude of factors associated with the incidence of seclusion and restraint that have the potential to be triggered during emergencies and global crisis situations, impacting in turn the rights of an already vulnerable population.

## 1 Introduction

In the context of mental health hospitals, restrictive practices, such as seclusion and restraint, may be indicated as minimal and last resort interventions intended to prevent imminent harm to the patient or others (1, 2). Despite this protective purpose, seclusion and restraint are inconsistent with human rights-based care and may lead to significant injuries as well as increases in morbidity and mortality (1, 3, 4). An international consensus was reached on the need to reduce restrictive practices. In response, governmental authorities from the Canadian province of Quebec released an updated reference framework in 2015 that provides essential guidance on coercion reduction and outlines the proper application of seclusion and restraint (5). However, COVID-19 pandemic-related restrictions have significantly changed the environment and the delivery of direct care in all health services, including psychiatric hospitals (6, 7).

Describing restrictive practices use is complex since definitions and medical records systems vary according to each country, which leads to heterogeneity in prevalence and incidence measurements (8–10). Over the last ten years, the prevalence of restraint use has declined: international studies indicate a prevalence between 5% and 16.4% (9, 11–15) but the range differences remain very variable, i.e. from 2% to 88% (16–19). The incidence of seclusion and restraint has remained poorly documented in Quebec over the last ten years, since ongoing monitoring through systematic collection is not subject to any provincial standard. The most recent study suggested that between 2007 and 2009, seclusion had been used on nearly a quarter (23%) of hospitalized patients in psychiatric care units, and mechanical restraint on 17.5% (20).

While any person experiencing a crisis situation while hospitalized in a mental health facility is likely to be subject to coercion, the use of seclusion and restraint seems more prevalent in men and in people with a psychotic or mood disorder (11, 12, 16). The growing body of research on the topic is demonstrating not only the ethical issues of limiting a person’s freedom, but also the many potentially harmful consequences for patients and organizations, such as risk of injury, deep vein thrombosis, mortality, distress, post-traumatic stress, perception of punishment, increased hospital stays, increased stigma, and loss of trust in caregivers (12, 21, 22). Considering this, the WHO (2, 23) has been standing up to defend human rights by calling for restrictive practices to be strictly limited to exceptional use and, in the longer term, entirely prohibited in order to support patient self-determination. The United Nations Rapporteur even identified them as a potential form of modern torture (24); however, a debate persists, named the Geneva impasse, where some advocate for their abolition, while others aim for minimal use (25).

Recently, the World Psychiatric Association adopted a position statement concerning the protection of people living with mental disorders or disabilities and released a call to action within the community to ensure alternatives to coercion (26). Researchers around the world have been studying various effective means of reducing the use of seclusion and restraint. These alternatives include the Safewards Model, Six Core Strategies, Open Door Policies and the World Health Organization’s QualtyRights Initiative (26). The province of Quebec is also following international trends by implementing an exhaustive revised reference framework (5) based on the *Act Respecting Health Services and Social Services* (118.1) which requires that all health care institutions provide a specific care protocol and perform annual evaluation of restrictive practices. Furthermore, since 2012, an important legislative change to professional codes allows several professionals (nurses, psychologists, physical therapists (for restraint only), social workers, occupational therapists, criminologists, educational psychologists and physicians) to decide on the use of seclusion and mechanical/physical restraint. Although this regulation promotes professional autonomy, it raises questions about a potential increase on the incidence of restrictive practices and reinforces the need to closely investigate this topic.

Following the declaration of COVID-19 (SARS-CoV-2) as a health emergency in January 2020 and then as a pandemic two months later (27), several authors expressed concerns that mandatory preventive measures, including isolation to minimize the transmission of the virus, may have had an impact on the incidence of restrictive practices in mental health institutions (28, 29). In this regard, international research shows that the COVID-19 pandemic led to a reduction in the frequency and/or length of psychiatric hospitalizations (30–32). For seclusion and restraint rates, results were mixed; some studies indicated a decrease (33, 34), while others an increase (35, 36). In Ontario, a study including 71 000 general adult hospitalizations showed a negative impact from the COVID-19 pandemic, where physical restraint use increased following public health restrictions (37). Less is known about the situation in Quebec, where the restrictive measures on the population were especially severe during the pandemic.

Almost 10 years after the study published by Dumais et al. (20), and since the implementation of governmental initiatives in 2015 to reduce the incidence of seclusion and restraint, there is no recent evidence from the province of Quebec that assesses the evolution of restrictive practices. Further, with the significant environmental disruptions related to the COVID-19 pandemic, it seems essential to examine its impact on the incidence of these practices. Thus, the aim of the present study is two-fold: 1) to retrospectively assess the incidence of seclusion and mechanical restraint in a Quebec mental health hospital over 4 years; and 2) to assess the impact of the COVID-19 pandemic on their incidence. In the wake of a global pandemic, it is possible to hypothesize that most of the factors influencing seclusion and restraint use are affected. Therefore, we hypothesize that the pandemic led to increased seclusion and restraint use.

## 2 Method

### 2.1 Design

We conducted a retrospective study based on the medical records of a Quebec mental health hospital collected between April 2016 and March 2021. April 1st, 2020 was selected as the initial date for the post-test data because it represents the first whole month of the public health COVID prevention measures being applied in Quebec institutions.

### 2.2 Population and sample

As part of a group of institutions, the targeted specialized mental health hospital serves the entire population of Montreal and its surroundings, which is approximately 1.7 million inhabitants. Of those, 535,600 (26%) live in the same area as the hospital where 2234 confirmed cases of COVID-19 were declared in April 2020. Six healthcare units of the mental health hospital were included, representing a mental and physical care unit, three psychiatric units (general), a psychiatric intensive care unit, and a psychiatric emergency room. The mission and users’ profiles of these care units remained relatively unchanged over the past decade, which justified their selection. We excluded the inpatient unit for people living with autism spectrum disorder or intellectual disability because of their specific application of seclusion and restraint, which may differ from other units. Also, reflecting a shift towards recovery and integrated care, many units are now dedicated to community care. Accordingly, outpatient clinics and community care were excluded as they do not apply seclusion and restraint.

Due to the diversity of services offered in this hospital, patients with any mental health diagnose(s) were considered, including individuals with multiple conditions or under investigation.

Two samples were drawn from a population of 19 143 distinct adult inpatients (18 years and older) who were either hospitalized in a care unit or were occupying a bed in the emergency department of the specialized mental health hospital from April 1st 2016 to March 30th, 2021. Using a purposive method, the first sample comprised all patients who had been placed under seclusion and the second sample comprised all patients who were subjected to mechanical restraint. Three analysis frames were used. The first time of analysis, spanning from April 2016 until March 2019, witnessed the natural evolution of clinical practices following the implementation of the 2015 governmental framework. The second period of analysis, from April 2019 to March 2020, corresponds to the period leading up to the declaration of the COVID-19 pandemic (pre-COVID). The third period, from April 2020 to March 2021, is the period following the declaration of the pandemic (post-COVID).

### 2.3 Data collection

#### 2.3.1 Patient characteristics

We extracted from medical records each patient’s available data: age, sex, moment of the restrictive practice application (night [0:00 to 7:59]; day [8:00 to 15:59], evening [16:00 to 23:59]), type of care unit, diagnosis, and length of stay (days).

#### 2.3.2 Restrictive practices

In this study, the term *restrictive practices* includes two type of formal coercion used as last resort when a person constitutes a danger to themselves or to others: (1) seclusion and (2) mechanical restraints. *Seclusion* is defined as a measure used to restrict a person’s freedom by confining them to a closed space from which they cannot freely leave (5). *Restraint* is defined as a measure involving the limitation of a person’s movement by human or mechanical force, or deprivation of a means to palliate a disability (5). In the targeted care settings, *restraints* are used only in the seclusion room and consist of immobilizing the person’s wrists and ankles on a gurney with restraints (lap belt). The use of chemical restraints was not accounted for in the records and was thereby excluded in this study. Data were collected using the restrictive practices protocol log, a manually completed document that records the type of restrictive measure, as well as the frequency and duration of the use as declared by registered nurses.

#### 2.3.3 Statistical analysis

Three types of descriptive statistics were used throughout this study: frequency distribution (number, N), measures of central tendency (mean, %) and measures of variation (SD, change rate, %). For the first objective, the incidence represented the proportion of patients who experienced one or more application(s) of seclusion and restraint during each month (±30 days) or each year under study. The number of patients who underwent the measures was divided by the total number of inpatients hospitalized or occupying an emergency bed during the same time period. To compare the different profiles of patients who experienced seclusion and restraint, chi square tests were performed. For the second objective, Welch’s t-test was performed to retrospectively detect the trend prior to the COVID-19 pandemic and the variance in incidence. Rates of change were assessed for each month and year during the COVID-19 pandemic using the following formula: (Incidence February/Incidence January)-1)*100. The collected data were handed using R CoreTeam 4.0.0 version (38).

#### 2.3.4 Ethical considerations

The study followed the recommendations of the Tri-Council Policy Statement: Ethical Conduct for Research Involving Humans (39) and the study was approved by the local ethics committee (CEMTL n2021-2267).

## 3 Results

### 3.1 Sample characteristics

In total, 1373 patients were subjected to at least one instance of seclusion between 2016 and 2021, while some form of restraint was applied at least once to 880 patients in the same period. **Table 1** lays out the main information used in the study, including the characteristics of individual patients and their institutional context. It should be noted that a patient who remains hospitalized for multiple years will appear in all corresponding analysis periods.

**Table 1:** Sociodemographic characteristics of inpatients subjected to seclusion and restraint.

Characteristics	Seclusion (N = 1373)			Restraint (N = 880)		
Time	2016-19 (N = 876)	2019-20 (N = 193)	2020-21 (N = 304)	2016-19 (N = 559)	2019-20 (N = 151)	2020-21 (N = 170)
Gender	N (%)			N (%)		
F	215 (33.2)	72 (37.7)	73 (31.9)	143 (33.6)	55 (39.9)	41 (28.5)
M	433 (66.8)	119 (62.3)	156 (68.1)	283 (66.4)	83 (60.1)	103 (71.5)
Chi^2^	73.34***	11.57***	30.08***	46.01***	5.68*	26.69***
Age	N (%)			N (%)		
18-34 y-o	122 (43.7)	14 (29.8)	104 (46.0)	91 (47.6)	13 (33.3)	74 (52.1)
35-44 y-o	65 (23.3)	16 (34.0)	48 (21.2)	45 (23.6)	13 (33.3)	30 (21.1)
+45 y-o	92 (33.0)	17 (36.2)	74 (32.7)	55 (28.8)	13 (33.3)	38 (26.8)
Chi^2^	17.48**	0.298	20.85***	18.39***	0	23.21***
Principal diagnosis	N (%)			N (%)		
Personality disorder	36 (5.6)	17 (8.9)	15 (6.6)	28 (6.6)	16 (11.6)	8 (5.6)
Schizoaffective	69 (10.6)	20 (10.5)	26 (11.4)	39 (9.2)	15 (10.9)	16 (11.1)
Other specified schizophrenia spectrum and other psychotic disorder	151 (23.3)	34 (17.8)	62 (27.1)	93 (21.8)	23 (16.7)	43 (29.9)
Mood disorder	91 (14.0)	33 (17.3)	32 (14.0)	60 (14.1)	20 (14.5)	19 (13.2)
Schizophrenia	108 (16.7)	29 (15.2)	29 (12.7)	65 (15.3)	19 (13.8)	17 (11.8)
Behavioral disorders	26 (4.0)	7 (3.7)	12 (5.2)	24 (5.6)	4 (2.9)	12 (8.3)
Mental impairment	25 (3.9)	11 (5.8)	5 (2.2)	19 (4.5)	9 (6.5)	2 (1.4)
Chi^2^	3316.8***	469.09***	629.46***	1712.2***	255.62***	321.06***
Length of stay	N (%)			N (%)		
<1 day	391 (44.6)	71 (36.8)	173 (56.9)	283 (50.6)	67 (48.2)	106 (58.2)
1 day	61 (7.0)	12 (6.2)	20 (6.6)	31 (5.5)	5 (3.6)	10 (5.5)
2 days	27 (3.1)	7 (3.6)	12 (3.9)	22 (3.9)	5 (3.6)	9 (4.9)
3 days or more	397 (45.3)	103 (53.4)	99 (32.6)	223 (39.9)	62 (44.6)	57 (31.3)
Chi^2^	562.08***	135.35***	225.92***	380.27***	102.24***	140.33***
Time of event	N (%)			N (%)		
Day	375 (42.8)	75 (38.9)	140 (46.1)	245 (43.8)	57 (41.0)	81 (44.5)
Night	140 (16.0)	35 (18.1)	53 (17.4)	76 (13.6)	21 (15.1)	23 (12.6)
Evening	361 (41.2)	83 (43.0)	111 (36.5)	238 (42.6)	61 (43.9)	78 (42.9)
Chi^2^	119.02***	20.56***	38.73***	98.13***	20.95***	35.15***
Chi^2^	650.9***	155.78***	335.63***	370.06***	105.96***	247.76***

*p < 0.05; **p < 0.01; ***p < 0.001.

On an individual basis, a larger portion of men were subjected to both seclusion (p<0.001) and restraints (p<0.05) throughout all analysis periods; women represented on average only 30-40% of cases. There is no significant difference between the age of the patients and the use of restrictive measures during the pre-pandemic second period of analysis, but for the first and third periods the youngest group of patients, aged between 0 and 34 years, were more often subjected to seclusion and restraints than patients between 35 and 44 or beyond 45 years of age. The patient’s diagnosis represents an important variable for which statistically significant differences between subgroups were observed in every analysis period (p<0.001). Specifically, seclusion and restraints were most frequently used in the case of patients suffering from non-organic psychosis (16-30% of cases), while they were least often used on patients presenting with an intellectual impairment (0-6.5%). Pertaining to the length of stay, the large majority of first episodes leading to seclusion or restraints took place in the first 24 hours (for example, in emergency settings) or after more than 3 days of hospitalization.

Looking at the time of the event, patients were generally less often placed in seclusion and restraints during the night shift than during the day and evening shifts (p<0.001). Finally, Psychiatric ICU and Psychiatric Emergency were the care units in which the largest number of patients were in seclusion or restraints, representing more than 70% of the total sample.

### 3.2 Incidence

#### 3.2.1 Seclusion

##### 3.2.1.1 Annual Incidence of Seclusion

In total, 1373 patients were noted as having been put in seclusion at least once from 2016 to 2021. The incidence during the first analysis period (2016-2019) was 4.17%, and this percentage remained relatively stable for the second period leading up to COVID-19 (2019-2020), rising only to 4.60%. A notable increase was reflected, however, in the data from third period following the declaration of the pandemic, with the annual incidence rising to its highest value, 14.1%, during the 2020-2021 period. The annual incidence of seclusion from 2016 to 2021 is presented in **Table 2**.

**Table 2:** Annual incidence of seclusion from 2016 to 2021.

Period	Year	Occupied Beds	Cases	Incidence (%)	Annual change (%)
Time 1	2016-17	3684	171	4.64	–
	2017-18	3825	151	3.95	-15
	2018-19	3963	156	3.94	-0.28
Time 2 pre-COVID	2019-20	4367	201	4.60	16.9
Time 3	2020-21	2184	307	14.1	205
post-COVID	2021	1120	98	8.75	-37.8

**Figure 1:**
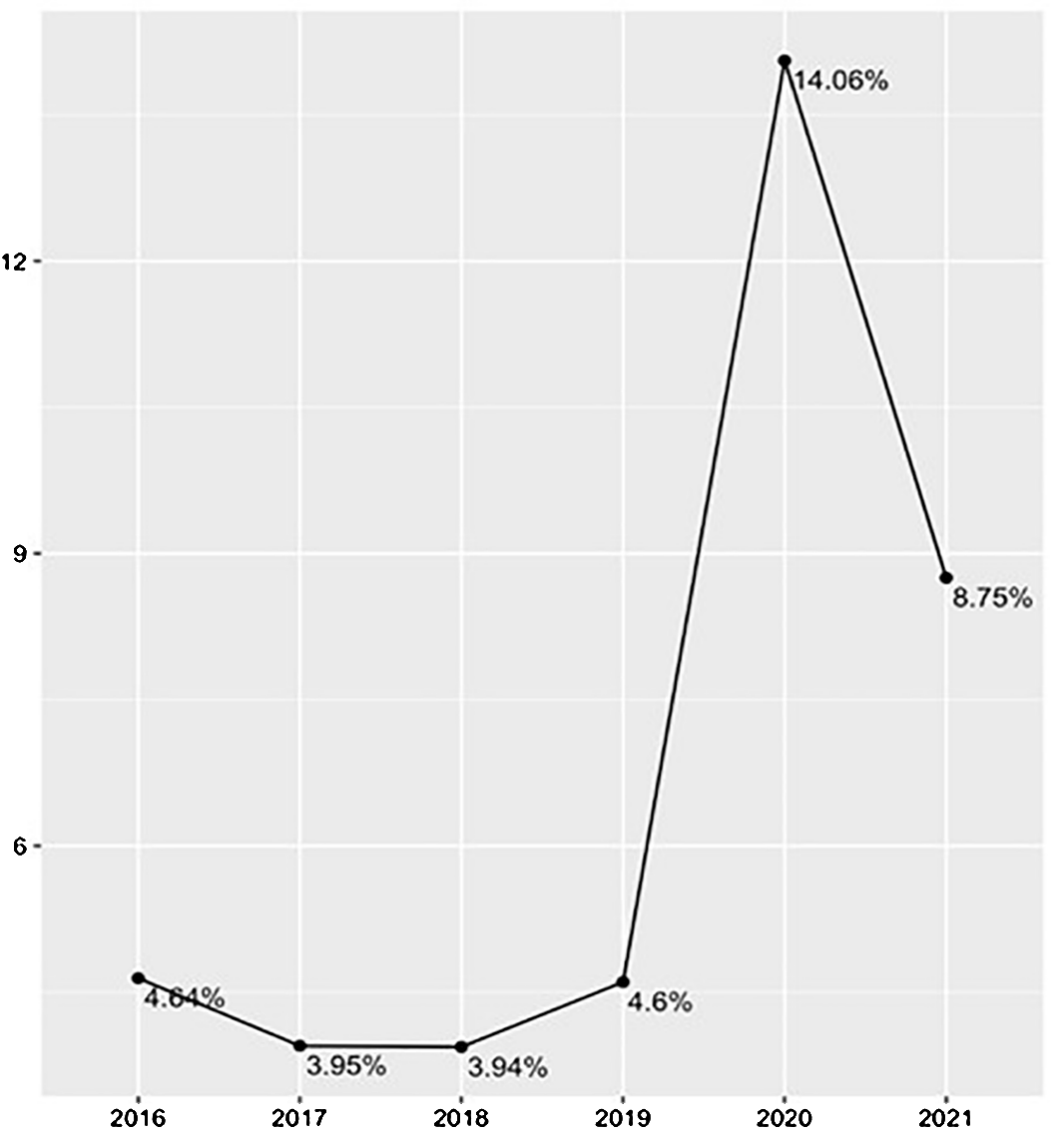
Annual Incidence of Seclusion from 2016 to 2021.

The increase in the incidence of seclusion noted between the period leading up to the declaration of the pandemic and the first year following it represents an augmentation of 205%. Welch’s t-test of independent samples demonstrated that the difference between these two analysis periods was statistically significant: t(17.27)=7.45, p<0.0001, IC95% (2.0;3.6). **Figure 1** shows the curve in the annual incidence.

##### 3.2.1.2 Monthly Incidence of Seclusion pre and post COVID-19 pandemic declaration

**Figure 2:**
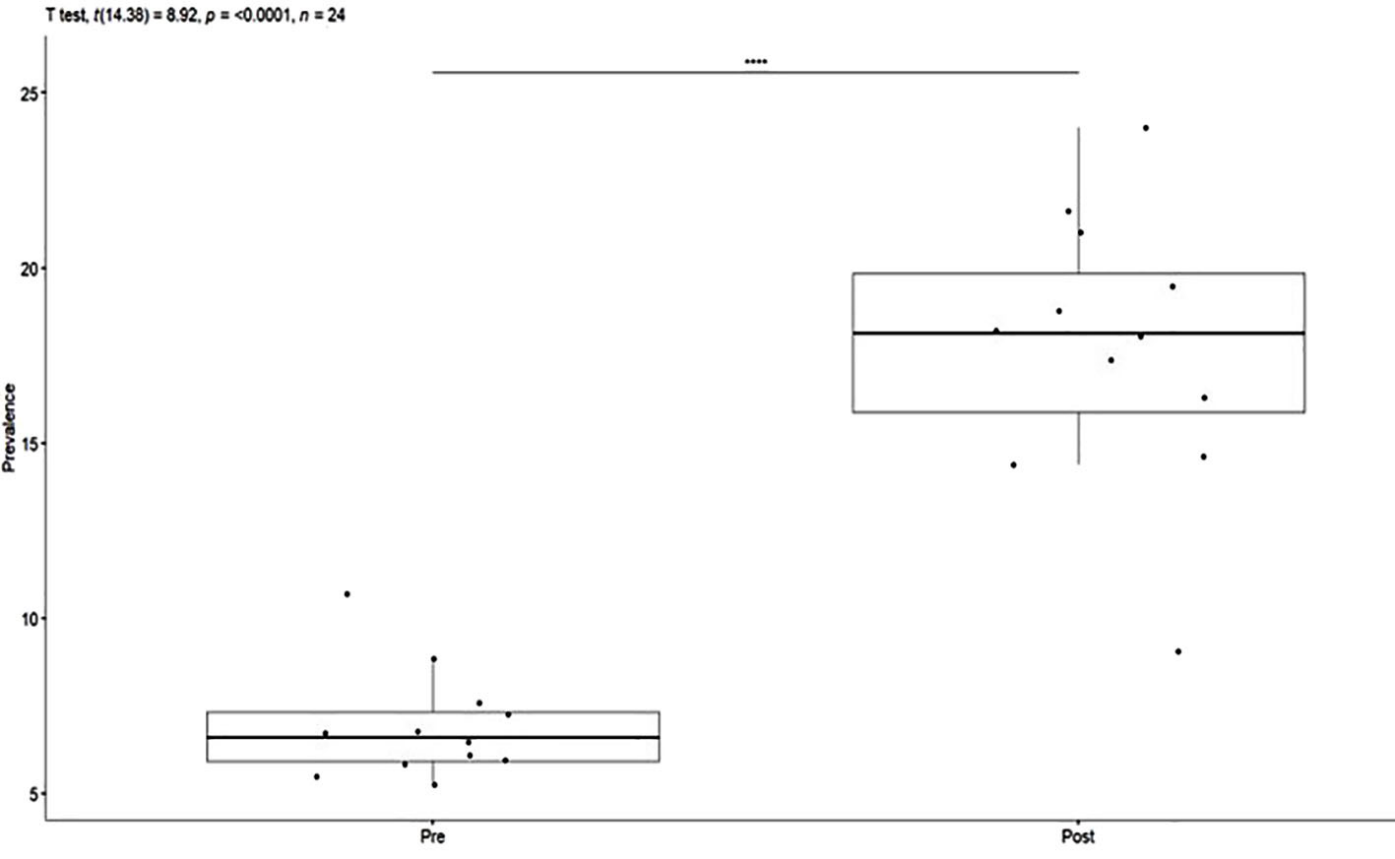
Monthly Incidence (%) of Seclusion for the pre-post COVID-19 periods.

The average monthly incidence of the seclusion measures pre-COVID-19 was 6.92% (SD = 1.55) and 17.7% (SD = 3.91) post-COVID. Welch’s t-test of independent samples demonstrated that this difference was statistically significant t(14.4)=8.92, p<0.0001, IC95% (8.2;13.4). Monthly incidence of seclusion for the pre-post COVID-19 periods are presented in **Figure 2**.

#### 3.2.2 Restraints

##### 3.2.2.1 Annual Incidence of Restraints

Over the course of this study, from 2016 to 2021, 880 patients were restrained on at least one occasion as shown in **Table 3**. The incidence during the first period (2016-2019) was 2.43%, then 3.18% during the second (2019-2020). Following the declaration of the pandemic, it rose notably during the third period to arrive at its highest value, 8.61%, in 2020-2021.

**Table 3:** Annual incidence of restraints from 2016 to 2021.

Period	Year	Occupied Beds	Cases	Incidence (%)	Annual Change (%)
Time 1	2016-17	3684	97	2.63	–
	2017-18	3825	89	2.33	-11.6
	2018-19	3963	93	2.35	0.86
Time 2 pre-COVID	2019-20	4367	139	3.18	35.6
Time 3	2020-21	2184	188	8.61	170
post-COVID	2021	1120	62	5.54	-35.7

**Figure 3:**
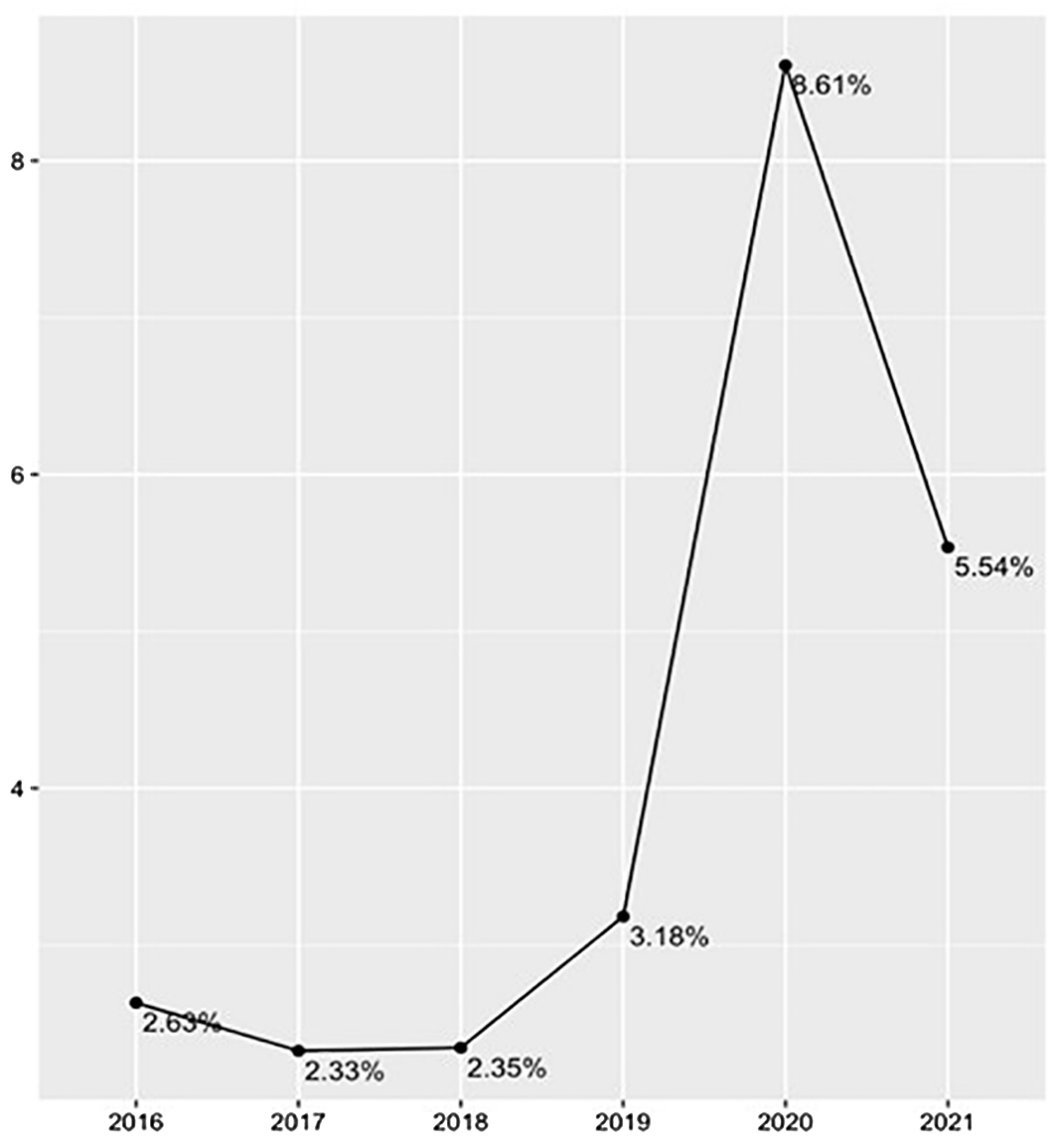
Annual Incidence of Restraints.

An increase of 170% for the use of restraints is demonstrated by the comparison of data collected before and after the declaration of the COVID-19 pandemic. Welch’s t-test of independent samples demonstrates that the difference between the two periods was statistically significant: t(17.6) = 3.97, p<0.001, IC95% (1.4;4.6). **Figure 3** shows the curve of annual incidence.

##### 3.2.2.2 Monthly Incidence of Restraints pre and post COVID-19 periods

**Figure 4:**
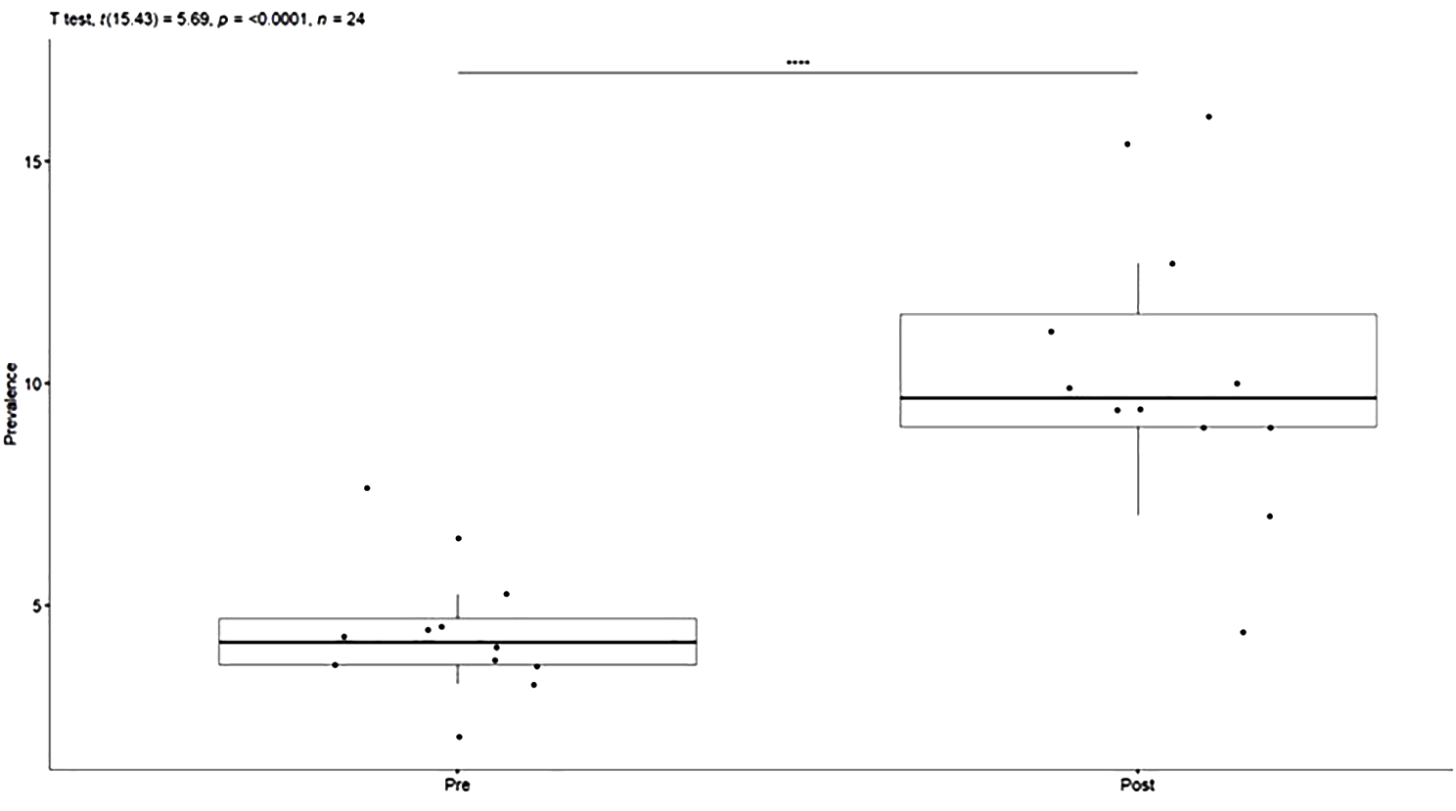
Monthly Incidence (%) of Restraints for the pre and post-COVID-19 period.

The average monthly incidence of the use of restraints before and after COVID-19 was respectively 4.42% (SD = 1.49) and 10.3% (SD = 3.24) as shown in **Figure 4**. Welch’s t-test of independent samples demonstrated that this difference was statistically significant t(15.4) = 5.69, p<0.001, IC95% (3.7;8.1).

## 4 Discussion

The aims of this study were to: 1) retrospectively assess the incidence of seclusion and mechanical restraint in a mental health hospital over 4 years and 2) to assess the impact of the COVID-19 pandemic on their incidence.

### 4.1 Incidence over a 4-year period

Preceding the COVID-19 pandemic onset, our results show that the incidence of seclusion and restraint was relatively stable over a 4-year period, with a non-significant decrease trend. Using the computerized database for seclusion and restraint in a specialized mental health hospital in Quebec, we found an average incidence of 4.17% for seclusion and 2.43% for mechanical restraint between 2016 and 2019. In comparison, the previous study conducted in the same region 10 years ago showed an average incidence of 23% for seclusion and 17.5% for restraint (20). Therefore, we observed a net decrease of nearly 10% for both seclusion and mechanical restraint, and a net reduction of approximately 80% which confirms our hypothesis indicating a decrease in restrictive measures since the governmental and institutional initiatives. However, this interpretation should be treated with caution, as the process of registering restrictive measures changed in 2015, and the units are not exactly the same. Nevertheless, results of the present study are also within the range of the majority of the most recent studies conducted in developed countries, which is between 5% and 16.4% (11–13, 15, 40, 41).

Changes in policies concerning the application of restrictive practices may also be responsible for the variation in incidence in industrialized countries as shown by Vruwink et al. (40), which demonstrates an increased incidence of seclusion after the end of a nationwide program of seclusion-reduction. In Quebec, several reorganizations of care policies surrounding the application and documentation of restrictive measures occurred in 2012 and 2015. A legislative change to Quebec’s Professional Code in 2012 mandated the recording of information related to restrictive measures in the patient’s record. In 2015, a significant transformation of the Quebec healthcare system led to the reassignment of nurses to new units and the transfer of patients to specialized units. Therefore, it is possible to see an association between the gradual decrease in incidence shown by this study and governmental and institutional regulatory initiatives to reduce the use of restrictive measures.

### 4.2 Before and after the pandemic onset

Our second aim, looking at the specific results related to the COVID-19 pandemic, shows an increase of 205% for seclusion and 170% for restraint. Our results confirm our hypothesis regarding the increase in the use of coercive measures during the pandemic, i.e., that there is a strong association. However, we cannot establish a causal relationship. In the literature regarding the impacts of the COVID-19 pandemic on the use of coercive measures, there is observable heterogeneity: some authors showed increased use of restraints (35, 36), while others showed a reduction in both restraints and seclusion (33, 42).

In light of these results, we propose examining the changes caused by the COVID-19 pandemic in relation to the 6 key domains identified in Bowers’s Safeward Model (43), which provides a comprehensive synthesis of the factors influencing conflicts and containment on psychiatric wards. These six domains have potential to influence the use of restrictive practices: staff team, physical environment, outside hospital, patient community, patient characteristics, and regulatory framework.

#### 4.2.1 Staff team domain

Staff modifiers were significantly impacted by the pandemic, particularly concerning contamination-related risks, uncertainty, fatigue, anxiety, and grief (44). There was an increase in demands for sick leave, leading to a growing need for backup staff who were less trained to manage crisis situations (44). The many sick days, new staff, and care teams not used to working together may have affected technical mastery, defined as “the depth and quantity of social and interpersonal skills and responses to patient challenge” (43). It is possible to assume that the lack of competent staff and the exceptional procedures related to the pandemic affected the contact between healthcare professionals and service users, thus affecting the quality of the therapeutic relationship. Since staff showed a high level of stress and anxiety, their capacity to regulate their normal emotional responses had the potential to be impaired. They may have been less caring and showed less humanity in usual situations of conflict or moral conflict when facing a patient refusing to apply preventive measures such as vaccination, wearing a mask or social distancing (45). Moral conflicts, such as the tension between the awareness that some users have a limited capacity to understand and apply sanitary measures and the risks associated with the spread of the virus, may also have influenced the staff’s psychological understanding for the difficult behavior of patients (28, 29). This leads to the question: were staff emotionally available and competent to deal with the complexity of the situations? In this regard, a Canadian study (34) conducted during the first few months of the COVID-19 pandemic showed a more than 50% decrease in seclusion after implementing measures to ensure patient respect, increase staff engagement in institutional procedures and provide the careful explanation of isolation measures.

#### 4.2.2 Physical environment domain

Caregivers faced two main challenges during the pandemic: the specific layout of the mental health care units, as their large proportion of common areas are environments where the risk of transmission is high, and isolation for quarantine purposes. Unlike other specialized care units, psychiatric units are environments where social contact between patients and staff is suggested and encouraged as therapeutic measures (28). Consequently, a significant environmental reorganization was carried out to reduce the risk of transmission for patients and medical staff; namely, patient were often sequestered in their rooms.

When patients have to stay in their rooms or quarantine, it is more complex for staff members to provide continuous supervision and support patients with early interventions. Furthermore, some units where the patients’ ability to comply with the sanitary measures was questioned were locked, most outing rights or temporary leaves were suspended, and outdoor activities (for example, access to the gym or daily walks) were cancelled, restricting patients to a closed physical environment. Therefore, the patient’s freedom was limited, and there could have been an increase in loneliness and boredom, as well as a lack of supervision, which are flashpoints of the physical environment domain.

#### 4.2.3 Outside hospital domain

Triggers coming from outside the hospital can originate from visitors and family tension, or the lack of support as expressed by the patient. The restriction on family visits to decrease the risk of transmission within the community could have potentially been positive or negative, depending on the nature of the relationship. Bad news from the outside, such as media coverage of the daily contamination rate and mortality attributable to COVID-19, can be anxiety-provoking, thus generating distressed and aggressive behaviors (46).

#### 4.2.4 Patient community domain

Although socializing with peers is usually encouraged in a therapeutic context, close contact, or any activity where patients must interact with each other, can be the starting point for conflict and are highlighted as flashpoints for this domain. During the COVID-19 pandemic, there were efforts to encourage social distancing, thereby reducing social density, which could have contributed to a decrease in the incidence of coercive practices. In this regard, the Feeney et al. (42) study, which reported a decrease of more than 50% in seclusion and restraint in the context of the COVID-19 pandemic, suggested that these results might be influenced by the diminution in contact between patients.

#### 4.2.5 Patients characteristics domain

During the pandemic, there was a desire on the part of healthcare institutions to reduce hospitalizations in order to limit the risk of transmission. Patients with increased severity of psychiatric symptoms were most likely to be hospitalized (44). The psychiatric vulnerability of inpatients can alter their ability to comply with preventive measures, including isolation, social distancing, and the use of masks (46), in addition to increasing the incidence of conflicts between users. Further, the deprivation of rights related to the cancelation of outings or temporary leaves can also create conflicts and aggressive behaviors, which could have impacted coercive measures (48).

#### 4.2.6 Regulatory framework domain

One of the hypotheses that can explain the increased in seclusion use is the oftentimes confusing distinction between isolation and seclusion. In Quebec, preventive isolation was required for any patient coming from another hospital or an intermediate resource, and screening tests were requested on several occasions during isolation (28, 47, 49–51). In the emergency room, as patients generally come from their homes or an intermediate resource, preventive isolation has been implemented extensively, which may explain the higher incidence of restrictive practices in this unit (47–49). For all inpatients on the unit, protective masks and social distancing had to be maintained at all times (47, 49). For patients who were positive or deemed unable to comply with the measures in place, isolation was also required (45, 47). This was also the case for some hospitals in the United States, Italy, Scotland and China, where they implemented preventive isolation measures to prevent an outbreak (28, 50, 52, 53). By definition, isolation and seclusion are the same thing, i.e., withdrawing a person by confining them in a specific area without the possibility of leaving freely. The difference between the two lies in the aim of the intervention, i.e., prevention of a risk of transmission for isolation versus prevention of aggressive behavior for seclusion, which can lead to confusion about how information is recorded in the service user’s file. For the service user who experiences them, there is also a concern related to the possible misinterpretation of the intervention and a risk of (re)traumatization.

#### 4.2.7 Looking forward

The previous sections critically examine several factors that may have influenced the increased restrictive practices we observed during the COVID-19 pandemic. To our knowledge, no data are available yet on the subsequent phases of the pandemic (2022) or the post-pandemic period (2023-2024) to assess its possible long-term impact on coercion. However, current trends point towards a continued decrease in restrictive practices, with a recent systematic review indicating a significant decrease in restrictive practices over the past ten years in North America, Europe, and Australia (54). This reflects a global commitment to alternatives to coercion and to support developing countries in elaborating and implementing alternatives consistent with their realities (55, 56). We therefore hope that future data will confirm this downward trend.

### 4.3 Limitations

This study has some limitations. First, while the validity of the data collected is supported by the completion of a mandatory and computerized protocol, some data may be under-reported or missing. Secondly, the use of chemical restraints is not documented in the protocol, so its incidence was not included in this study. Thirdly, the computerized protocol did not allow for reliable documentation of the duration of seclusion or restraint. This information was therefore not included in the study. Finally, we recognize that, as we did not have access to data on the incidence of restrictive measures in 2022 and 2023, the current portrait of incidence could be incomplete. Given that this study was conducted in a single specialized mental health facility, results may not be transferable to hospitals where the protocols put in place by the government to address the pandemic differ. Finally, the use of non-parametric tests was justified by abnormal distributions and small sample sizes.

## 5 Conclusion

Analyzing the significant increase in seclusion and restraint use after the COVID-19 pandemic onset according to the six domains of Bowers’s Safewards Model (43) allows us to take a new perspective on the consequences of a pandemic for inpatient mental health settings. Beyond the fact that it is never a single factor that explains statistical variations over time, such as those observed in the use of seclusion and restraints, the COVID-19 pandemic had a particularly significant potential to trigger several flashpoints associated with conflicts in mental health settings. The debate over COVID protection policies exaggerates the tension between autonomy and safety that is always present on a psychiatric unit (44). In crisis situations, the essential priority must be the management of the crisis itself, and this can restrict the attention that is given to other issues in an overall context of limited human resources. It is therefore possible to believe that this situation would lead to a shift in the aims of the organizational leadership from the reduction of restrictive measures towards a reduction of the transmission of infections. Recognizing that there has been an increase in the use of seclusion and restraints during the COVID-19 era, moving forward how can we prepare the healthcare system in order to ensure that clinical practices respectful of human rights will be maintained during any new crisis of this scope?

## Data Availability

The data analyzed in this study is subject to the following licenses/restrictions: Datasets are owned by the institution where the study was carried out. Requests to access these datasets should be directed to marie-helene.goulet@umontreal.ca.
